# A Survey of the Challenges Faced by Individuals with Disabilities and Unpaid Caregivers during the COVID-19 Pandemic

**DOI:** 10.3390/ijerph191610075

**Published:** 2022-08-15

**Authors:** Yashoda Sharma, Alison Whiting, Tilak Dutta

**Affiliations:** 1 KITE Research Institute, Toronto Rehabilitation Institute—University Health Network, Toronto, ON M5G 2A2, Canada; 2 Institute of Biomedical Engineering, University of Toronto, Toronto, ON M5S 3G9, Canada

**Keywords:** disabilities, unpaid caregivers, COVID-19 pandemic, changes in access

## Abstract

The COVID-19 pandemic negatively affected many individuals. In particular, it is likely that individuals with disabilities and unpaid caregivers were disproportionately affected, however, its exact impact is largely unknown. The primary objective of this work was to identify challenges faced by individuals with disabilities and unpaid caregivers. A secondary objective was to identify potential solutions to the major challenges experienced by both populations. Two surveys were administered online to individuals with disabilities and unpaid caregivers, respectively between September 2020 and January 2021. We used an inductive thematic analysis within an interpretivist paradigm to analyze survey responses. A total of 111 survey responses were collected amongst both surveys. Separate thematic maps were created for individuals with disabilities and unpaid caregivers, and maps were drawn to compare challenges. Potential solutions to mitigate the challenges experienced by both populations include revising financial assistance programs and improving awareness of support programs that are available.

## 1. Introduction

Individuals with disabilities and unpaid caregivers make up a large portion of the Canadian population. In 2017, 6.2 million Canadians 15 years of age and older had a disability [1], which when compared to the 4.4 million Canadians of all ages with disabilities in 2006 [2], highlights the increasing requirement to address the needs of this growing population. In recent years, nearly half of all Canadians with disabilities received support from another person to complete everyday tasks [3] such as household chores, which is often provided by unpaid caregivers. Unpaid caregivers provide both emotional and physical support to a friend or a family member with a medical condition or disability, including helping with transportation, household chores, and finances [4]. In 2015, unpaid caregivers provided care that saved Canadian care systems (e.g., health, social and community) CAD 24–31 billion [5]. For context, it was anticipated that nearly CAD 265 billion would be spent on Canadian health care in 2019 [6]. Additionally, 7.8 million youth and adult Canadians acted as unpaid caregivers in 2018 [7], with the average unpaid caregiver providing nearly 20 h of week of caring duties [8].

Prior to the COVID-19 pandemic, both individuals with disabilities and unpaid caregivers faced many challenges in their day-to-day lives. We know individuals with disabilities have experienced challenges accessing health care services (e.g., rehabilitation), partly due to financial constraints and limited access to transportation [9]. Beyond challenges with accessing healthcare services, Canadians with disabilities experience reduced employment rates and have a lower income compared to the rest of the population [1], thus increasing their chance of living in poverty. Similarly, unpaid caregivers are known to experience mental health concerns (e.g., distress and depression) [10,11] and mental and physical burnout [12], with greater than one in three unpaid caregivers in Canada reporting distress [13]. These individuals also expressed financial concerns such as a lack of government assistance [7]. It is expected that the COVID-19 pandemic magnified these challenges; however, its impact remains largely unknown.

Canada declared its first state of emergency on 23 March 2020 [14], and a series of protective measures were implemented to contain the virus. These measures included closures of non-essential workplaces, businesses, schools, and childcare centers, in addition to disruptions to health care services. Since the start of the pandemic, preliminary research has shown that individuals with disabilities faced a multitude of challenges. For example, this population experienced difficulties with accessing healthcare and COVID-19-related information, job security, and a loss of social connectedness [15,16,17,18]. Amongst other elements, individuals with disabilities faced physical decline, mental health concerns (e.g., isolation) and financial constraints largely because of the lack of access to the services mentioned previously. Additionally, two scoping reviews synthesized the literature on the health and social impacts and barriers to social engagement during COVID-19 for individuals with disabilities [19,20]. Overall, it appears the challenges individuals with disabilities faced prior to the pandemic were magnified. Interestingly, the exacerbations of pre-pandemic challenges were not unique to individuals with disabilities, but rather they were also experienced by unpaid caregivers. Although the magnitude of challenges differed between both population groups, up to 40% of unpaid caregivers reported having a worse time providing care during the pandemic [21]. Beyond additional worries of their loved one becoming infected by the COVID-19 virus from a paid caregiver [22], in some cases unpaid caregivers felt they needed to increase their caregiving load because of a lack of caregiving support (e.g., friends, family, formal respite care) due to financial constraints, or in fear of transmission of the virus [23]. Additionally, unpaid caregivers were faced with managing more complicated medical tasks from home [24]. To this end, unpaid caregivers were fatigued and burnt out [23,25].

Although there has been some initial research into this topic, the impact of the COVID-19 pandemic on individuals with disabilities and unpaid caregivers is emergent. Of the existing literature, many studies focus on specific disability groups, such as individuals with intellectual disabilities [15,18,26,27], which fail to give readers a comprehensive understanding of the pandemic impact on other disability groups. Additionally, to our knowledge, no other study has investigated the impacts of the COVID-19 pandemic on individuals with disabilities and unpaid caregivers in tandem. This is of great interest because it allows us to bring to light factors that have had the greatest impact on both groups, ultimately better supporting potential solutions. Finally, a noticeable number of papers haven been written outside of a Canadian context, which will not support recommendations to government and policies in Canada. Therefore, the primary objective of this work was to identify challenges faced by individuals with disabilities and unpaid caregivers, with a particular focus on understanding how changes to access to healthcare services (e.g., appointment cancellations, online/virtual appointment) has impacted individuals with disabilities and how the caregiving burden has changed for unpaid caregivers. As previously stated, simultaneously studying the impact of the pandemic on both population groups allowed us to identify potential solutions that will result in the greatest positive impact for these population groups that can be addressed by future research, government, and policies.

## 2. Materials and Methods

This was a qualitative research study. We grounded our work within an interpretivist paradigm and analyzed the data using an inductive thematic analysis [28]. With this work, we sought to understand and describe the meaning of the lived experiences of individuals with disabilities and unpaid caregivers.

There is no singular definition of disability, however, for this study, we included anyone who self-reported as having a medical or health condition (e.g., visual impairment) that impacted their capacity to participate in society. Unpaid caregivers were defined as individuals who provided care for a person with a disability (usually a family member, friend, or neighbor) without being compensated. All participants were 18 years of age or older and living in Canada. Online surveys were administered to individuals with disabilities and unpaid caregivers between September 2020 and January 2021.

Subject recruitment and participation in the study occurred entirely online and all survey responses were anonymous. Both surveys consisted of open- and closed-ended questions. Closed-ended questions were focused on participant demographics (e.g., age, gender, income, and employment), severity and type of disability (if applicable), and risks and challenges of COVID-19 (e.g., diagnosis of COVID-19, risk of contraction, risk of becoming seriously ill, and impact on individual and household). Some questions related to disability (e.g., functioning) were adapted from the Brief Model Disability Survey created by the World Health Organization [29]. Open-ended questions focused on the impact of COVID-19 on the lives of respondents and included four foci: housing, employment, finances, and emotional well-being. Some questions differed between surveys to better highlight the challenges faced by each population group. Examples of open-ended questions for individuals with disabilities included “If you caregiver gets COVID-19, what items do you have access to in order to protect yourself from getting COVID-19”, “Please describe how COVID-19 has affected your housing (i.e., needing to move in with your caregiver, family member, friend or vice versa)”, and “Please describe how COVID-19 has affected your emotional well-being (i.e., increased stress or anxiety)”.

A six-phase process was followed to analyze the survey results: “(1) familiarization with data, (2) generalizing initial codes, (3) searching for themes, (4) reviewing themes, (5) defining and naming themes and (6) producing the report” [28]. Following the inductive nature of this analysis, responses to survey questions were analyzed without taking into consideration the question that was asked. The results of each survey were analyzed independent of one another; therefore, this six-step process was performed twice.

Two members of our team (YS and AW) independently performed phases 1 and 2. A coding guide was created based on the generated list of preliminary codes developed by both members. The development of the coding guide was iterative, with updates being made throughout the analysis process. Coding disagreements were resolved via consensus decision making. The analysis and theme development commenced after there was complete agreement on the coding for each respective survey. To ensure trustworthy results, YS and AW kept memos throughout the individual coding process and met regularly to discuss thoughts and personal assumptions made when coding. For transparency, personal assumptions that were made throughout the analysis are described in the remainder of this paper. Finally, to ensure credible results, we provided full sentences and thick descriptions of participant responses [30]. The remaining phases (3 to 6) were completed collaboratively. The identification and refinement of themes was undertaken iteratively. We created separate thematic maps for each survey and later compared them to one another to gain a deeper understanding into the similarities and differences of experiences between individuals with disabilities and unpaid caregivers.

## 3. Results

### 3.1. Participant Characteristics

A total of 111 participants completed the surveys, including 68 participants who completed the individuals with disabilities survey and 43 participants who completed the unpaid caregivers survey. Descriptive statistics for our two groups of participants are summarized in Table 1. Most participants were female in the individuals with disabilities survey and caregiver survey (77.2% and 85.7% respectively). Over 75% of participants in both surveys were between the ages of 31 and 70, with 76.2% in the individuals with disabilities survey and 85.7% in the unpaid caregiver survey. Most participants self-identified as “White or Caucasian” with 89.1% reporting this in the individuals with disabilities survey and 80.5% in the unpaid caregiver survey.

Level of functioning for individuals with disabilities is summarized in Table 2. More than half of our participants reported having extreme problems with joining community activities (52.3%). Just under half of participants reported extreme problems with feeling tired and not having enough energy (48.4%). Over half of participants reporting having no problems with activities of daily living such as toileting (56.9%) and getting cleaned and dressed (18.46%). A wide-range of medical diagnoses were shared, and included those that impacted participants’ sensory, physical, cognitive, and mental health abilities.

**Figure 1:**
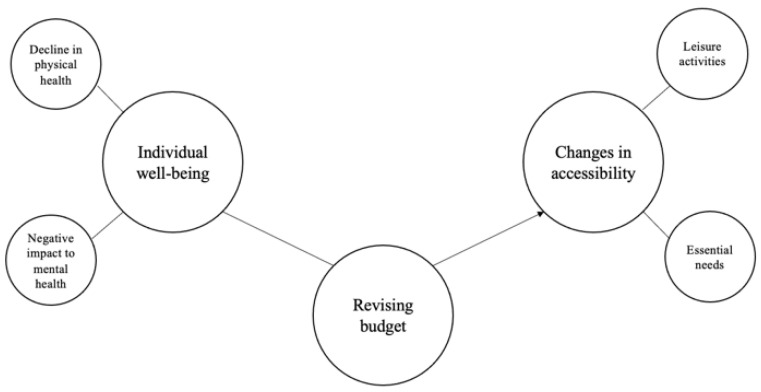
Individuals with disabilities thematic map. Large cells represent main themes and small cells represent sub-themes. The arrows represent the relationship between themes.

**Figure 2:**
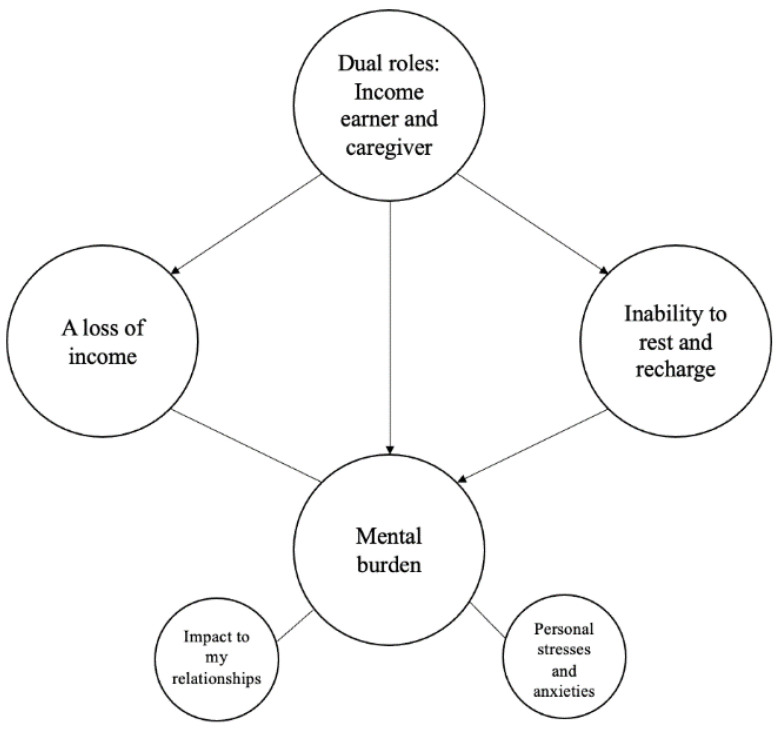
Unpaid caregiver thematic map. Large cells represent main themes and small cells represent sub-themes. The arrows represent the relationship between themes.

We developed two thematic maps to represent the ideas and concepts described in each survey. The thematic maps can be seen in Figure 1 and Figure 2. Some themes were related to others, as represented by arrows.

### 3.2. Individuals with Disabilities

We discovered three main themes, as shown in Figure 1: (1) individual well-being, (2) changes in accessibility, and (3) revising budget. Six participants did not contribute to the long answer questions used in this qualitative analysis. Table 3 displays quotes from individuals with disabilities that developed each theme.

#### 3.2.1. Individual Well-Being

The first theme developed was related to well-being, which encompassed two sub-themes: decline in physical health and negative impact to mental health. The cause of these declines in physical and mental health were multifaceted, however, they both involved a similar sequence: a change in or lack of routine, or fear of contracting the virus, caused a more sedentary and lonelier lifestyle, which then resulted in declines in physical health (e.g., increases in pain, loss of energy) or mental health (e.g., anxiety or depression).

**Decline in physical health.** A share of 37.10% of participants commented on their physical health. The pandemic negatively impacted participants’ physical health, to the extent that it began to hinder participants’ abilities to perform basic and essential activities of daily living, such as getting dressed or brushing their teeth.

As previously noted, reasons for these feelings were often because of a change in or lack of routine, or fear of contracting COVID-19. This change in or lack of routine looked different for everyone—examples include changes to accessing healthcare services, or changes to their everyday activities. The fear of contracting the virus stopped participants from doing simple activities such as taking walks, or accessing services that were available (e.g., medical tests), ultimately contributing to worsening physical health.

At the time of this survey, some healthcare services such as family physicians and physical therapy appointments were being offered virtually. Although participants did not explicitly report accessing virtual healthcare services, apart from physician appointments, it remained unclear whether participants were *choosing* not to access these virtual services, or if they were *unable* to access them because of barriers they encountered/perceived.

**Negative impact to mental health.** A total of 95.16% of participants expressed an impact of COVID-19 on their mental health. Participants expressed feelings of depression, anxiety, and stress from being socially isolated and concerned with contracting COVID-19. Participants were struggling with the fallout of not being able to see their friends and family; however, none reported disobeying the COVID-19 guidelines set out by the governments. In fact, the worry and fear associated with contracting COVID-19 seemed to overpower their desire to connect with friends and family, leaving many participants confined to their homes. In not being able to socialize, we felt participants lost both their sense of community and sense of belonging such that, without their support systems, they appeared to be helpless and lost. For example, one participant reported being suicidal again because of the stress induced by the virus and, in another instance, a participant reported needing to use recreational drugs to manage feeling alone.

In analyzing survey responses, we felt the impact of the COVID-19 pandemic on mental health was more meaningful to individuals with disabilities than the impact on their physical health. Although participants faced physical decline, we felt the severity of mental health concerns raised by participants were a strong indication that they could not cope well with the social isolation and restrictions.

#### 3.2.2. Revising Budgets

A total of 74.19% of individuals with disabilities shared experiences related to their budgeting. This theme was borne from participants sharing experiences of increased expenses, such as the rising cost of food, and purchases of personal protective equipment and other cleaning supplies. These costs were not part of participants’ budget planning prior to the pandemic, which resulted in participants facing challenges with staying afloat financially.

In some cases, participants were also experiencing interruptions to their income. This was either because of their job becoming obsolete due to the pandemic, their wages being reduced, or their other sources of income (e.g., government assistance) being delayed.

Some participants did mention that the increased financial challenges were somewhat offset by certain expenses being reduced during the pandemic. For example, some commented that they were using their car less and saving money on school lunches for their children because of virtual learning.

It was evident that, in revising budgets, participants had to make decisions about what they could access. In particular, we could see that participants had challenges with food accessibility in large part due to rising costs of grocery essentials. Thus, we noted a relationship between the theme of Revising Budgets and Changes in Accessibility.

#### 3.2.3. Changes in Accessibility

Participants noted they had to come to terms with a new way of living that posed challenges. To understand specifically how these changes in accessibility impacted individuals with disabilities, this theme was divided into two sub-themes: *essential needs* and *leisure activities.*

**Essential needs**. A total of 90.32% of participants commented on the changes to their essential needs. Participants faced difficulties booking appointments with their healthcare providers throughout the pandemic. Some participants reported feeling like they needed to “fight” for each appointment. Others reported their appointments being cancelled or delayed. In one case, a physician requested the participant book an in-person appointment; however, the participant reported challenges with getting through to the receptionist, stating “waiting 10 days for a phone call to get antibiotics is not good”.

Although in-person appointments were suggested to some participants, it was most common for participants to have virtual medical appointments. Interestingly, what appeared to be the biggest challenge amongst these participants was getting their needs met in virtual appointments. For instance, some participants reported that many physician appointments were performed over the telephone, which had some questioning the effectiveness of this service. It was hard for participants to remember to share all their ailments with their physician, and some had concerns about how well the physician could understand their situation without physically seeing their injury. Finally, with virtual medical appointments came the need for participants to have access to phones or other technologies, such as computers. This posed a challenge for some participants and highlights how virtual care can only support a segment of society.

Other notable areas where a change in accessibility was reported was food access and transportation. Participants were scared to visit grocery stores in person due to the crowds and risks of contracting COVID-19, and thus relied on their friends and families to support them. Additionally, participants raised concerns related to budgeting and food access, including delivery service fees and the rising cost of food, which they never previously budgeted for. This left a participant feeling that they could not afford to eat from all the food groups.

Similarly, participants reported avoiding public transit (e.g., buses) to avoid crowds. Thus, participants relied more on private transportation (e.g., rides from friends and family, taxis) where they felt more comfortable.

**Leisure activities**. A total of 66.13% of participants discussed the changes to their leisure activities. We felt that participants struggled to fill their time in meaningful ways. Participants reported a sense of boredom due to limited access to hobbies and activities of interest, and the changes in their relationships with friends and families.

This lack of activity seemed to translate into some participants feeling like they had lost their sense of purpose and/or belonging, which negatively impacted their quality of life. There were different reasons why participants could not take part in their usual leisure activities, including the concern of contracting COVID-19. The risk of contracting the virus impacted participants’ willingness to attend activities even when opportunities were open. Interestingly, a participant also commented on the benefits of COVID-19 to their leisure activities. This was a perspective to the pandemic that demonstrated how some people were able to take away a positive experience.

### 3.3. Unpaid Caregivers

We discovered four main themes when analyzing the responses to the unpaid caregiver survey: (1) a loss of income, (2) dual roles—income earner and caregiver, (3) inability to rest and recharge, and (4) mental burden.

Eight participants did not contribute to the long answer questions used in this qualitative analysis. Table 4 displays unpaid caregiver quotes that developed each theme.

#### 3.3.1. A Loss of Income

More than half of unpaid caregivers (65.71%) expressed changes in their income. This theme represents changes in working hours and subsequent income for unpaid caregiver participants. Participants associated their loss of income to two things: (1) economic impact of the pandemic, and (2) a change in their caregiver demands. This two-fold impact indicated that participants’ earning potential was impacted both directly and indirectly by the pandemic.

Although less common, some participants found their income was reduced or completely lost because of a direct economic impact of the pandemic. This was because participants experienced their places of work being shut down, or a lack of demand of the services they offer. Ultimately, this resulted in participants losing income.

Other participants were not explicit in why their income was lost or reduced; however, given the economic climate of the pandemic at the time data was collected, it is reasonable to assume that it was because of the business closures and lockdowns mandated across Canada [31].

Our findings showed the most common reason for a loss of income for participants was because of the personal impact of the pandemic. Participants reported needing to reduce their working hours to take on new or expanded caregiving duties because of limitations to alternate care options. For example, one participant reported needing to work around their loved ones’ day program, since there was no access to other care. We felt any flexibility participants had in managing different roles prior to the pandemic was now lost or reduced, and many participants had no choice but to take on a larger caregiving role and, consequently, have their incomes reduced.

It was evident that this theme was directly related to the following theme, dual roles—income earner and caregiver, because participants’ loss or reduction in income appeared to be directly tied to how well they could balance these two simultaneous roles.

#### 3.3.2. Dual Roles—Income Earner and Caregiver

Nearly half of participants (48.57%) expressed changes to their everyday roles. We identified a pattern in participant responses as they related to managing the roles of paid employment and unpaid caregiving. This theme highlights the many ways unpaid caregivers provide essential services in a community. It was apparent that caregivers were needing to switch their focus from being a primary income earner, to a primary caregiver. Participants were not necessarily stepping away from their paid employment *completely*, but rather were reprioritizing how to spend their time to manage their expanding responsibilities.

The overall workload of these participants increased because the respite care they relied on was no longer available or no longer considered safe. Examples of respite care that participants were missing were day programs, babysitters, personal support workers, and other caregivers. With this new reality came an inability for participants to rest and recharge, thus contributing to an increased mental burden.

#### 3.3.3. Inability to Rest and Recharge

More than half of unpaid caregivers (65.71%) expressed changes in their self-care routine and available time. We developed this theme based on participants reporting a lack of time to care for themselves due to the changing demands of their roles. For example, participants expressed little time to pursue hobbies of interest, leisure activities, or self-care, and instead were focusing more on their caregiving duties.

These examples demonstrate the challenges participants were experiencing trying to balance their expanded roles, while still finding time to care for themselves. It was clear that participants were unable to rest and recharge because of the limited supports they had, whether through paid community services or informal supports from their friends and family. Ultimately, this did have negative repercussions to their mental health.

#### 3.3.4. Mental Burden

While participants shared how their roles were changing and a lack of time for self-care activities, they were simultaneously describing how this impacted their mental health. The theme of mental burden describes participants’ feeling isolated from their loved ones, and stress and anxiety over their changing roles and the fear of contracting COVID-19. This theme was broken down into two sub-themes: *Impact on my relationships*, and *personal stresses and anxieties.*

**Impact on my relationships.** A total of 57.14% of participants had their relationships impacted by the COVID-19 pandemic. Participants could not develop or maintain connections with friends and family for fear of spreading COVID-19, lockdown restrictions, or because a lack of time. These missing relationships translated into a feeling of isolation and loneliness.

Although maintaining relationships amongst people outside of the household was challenging, some participants faced challenges within their households. Participants needed to stay home and could only socialize with their household members, causing strain to some family relationships. This demonstrates that, although the isolation was felt from not being able to socialize with the greater community, there were also feelings of tension because of the *increase* in socialization within the members of the home.

**Personal stress and anxiety.** One of the biggest challenges experienced by participants was related to their stress and anxiety, with over 80% of participants sharing these experiences (85.71%). There were many reasons for the stress and anxiety participants were experiencing, with the two largest reasons being caregiving demands and fear of the person they care for contracting COVID-19. Feelings of stress and anxiety and worrying about their loved ones were common, and some participants described their anxiety as being in “overdrive” or “at its max”. Other participants felt deflated and “bogged down” with their extra caregiving responsibilities.

Other reasons for the stress and anxiety were related to financial challenges and contracting COVID-19 themselves. For example, one participant shared how they were experiencing suicidal thoughts because of their concern with being able to financially support their child with disabilities.

## 4. Discussion

To our knowledge, this is the first study to take a combined approach to investigating the effects of the COVID-19 pandemic on individuals with disabilities and unpaid caregivers.

### 4.1. Individuals with Disabilities

We identified three major themes in the individuals with disabilities survey: (1) individual well-being, (2) changes in accessibility, and (3) revising budgets. Participants experienced declines in both their physical and mental health. However, we felt participants had a harder time managing the mental health repercussions caused by the social isolation and fear of contracting COVID-19. Participants lost their sense of community and felt helpless; in some extreme cases, participants expressed turning to recreational drugs or having suicidal thoughts. These findings agree with previous work that found that social isolation and loneliness can contribute to damaging effects to mental health [18,32]. Interestingly, the work done by Lake et al., 2021, also demonstrated participants’ resilience and willingness to identify other opportunities to connect with their loved ones (e.g., using Zoom and more telephone calls) to mitigate the impact of social isolation. However, these were findings that were not discussed by participants in the present study.

In our study, one of the most upsetting concerns for participants was related to the challenges they experienced accessing healthcare providers. Beyond the difficulty with booking appointments, participants reported most physician appointments were held via telephone. Many felt that they could not effectively communicate their concerns via telephone. Moreover, there was some indication that the disruption to allied health services (e.g., physical therapy) caused increases in pain for participants. Similar results are seen in another study [16], which also had a subset of participants who supported the opposite, where participants were “hoping for more virtual medical appointments” once the pandemic had subsided. Only 80% of individuals with disabilities in Canada use Internet services [3], as compared to the 91% of youth and adult Canadians [33]. Thus, we question whether virtual care encourages equal access to care for *all* Canadians. Participants also experienced access changes with respect to their leisure activities. It appeared participants had a sense of “boredom” since they did not have access to their activities of interest and were unable to fill their time in meaningful ways. Ultimately, this did play a negative role in participants’ happiness and emotional well-being.

Lastly, participants needed to revise their budgets, primarily because of their increased expenses. With the rising cost of food and the purchase of personal protective equipment and other cleaning supplies, participants were struggling financially. In contrast, a small number of participants found that certain expenses were reduced during the pandemic, such as money spent on school lunches and money spent on driving.

### 4.2. Unpaid Caregivers

We developed four themes when analyzing the unpaid caregiver survey: (1) a loss of income, (2) inability to rest and recharge, (3) dual roles—caregiver and income earner, and (4) mental burden.

Participants experienced a loss of income during the pandemic, which was mostly related to their increased caregiving demands. Participants reported reducing their paid working hours or work outside of their caregiving hours to accommodate their new responsibilities. Thus, there was a direct relationship to the second theme, dual roles—income earner and caregiver. In this second theme, participants highlighted how the pandemic required them to reprioritize their day to manage their expanding responsibilities (e.g., of a caregiver and income earner). These changes caused an increase in workload because they were unable to rely on other respite care services (e.g., babysitters). Similar results are seen in a study looking at the experience of stroke caregivers during the pandemic, where caregivers were spending more hours providing care because of respite service closures [24].

Participants were unable to rest and recharge, which contributed to their mental burden. Participants did not have enough time for self-care due to the time required to juggle their roles as an income earner and caregiver. This meant participants had less time for hobbies, or leisure activities, which negatively impacted their mental health. Further to this, participants were unable to keep connections with their friends and family because of the concern of transmitting COVID-19, lockdown restrictions, or simply because they had no time. This ultimately played a role in participants feeling isolated and lonely.

Participants also had increased stress and anxiety, primarily because of their caregiving demands and the fear of the person they care for contracting COVID-19. Similar results related to emotional and mental health concerns are found elsewhere [23,34]. Lightfoot et al., 2021 identified caregiving stress as a theme, based on participant feelings of social isolation, financial concerns, and managing the increased caregiving load while working. Other reasons for the stress and anxiety were related to financial challenges and contracting COVID-19 themselves.

### 4.3. Recommendations and Future Work

Based on the results of our study, we identified potential solutions that should be investigated in future research and may reduce the negative impact of future pandemics or emergency situations on individuals with disabilities and unpaid caregivers.

**Solution 1: Safe and continued access of respite care.** It was clear that the need for formal and informal supports (e.g., day programs, babysitters, personal support workers) were of the utmost importance for unpaid caregivers. However, these services were not used because of business closures and concerns with transmitting and contracting COVID-19. Hand hygiene has been recommended by the Canadian government to minimize the spread of COVID-19 [35]. Thus, it makes sense to identify strategies that can improve hand-washing practices at the level of the individual, to allow for safe social interactions and essential services to be more available. A potential solution for managing infection control rates can be hand hygiene systems in the home. In fact, 69.2% of unpaid caregivers (n = 39) and 67.2% of individuals with disabilities (n = 64) said they would want an alcohol dispenser in their home.

**Solution 2: Offering medical services in more ways than virtual.** Although virtual medical appointments offer patients the flexibility of taking calls from home and reducing the risk of COVID-19 transmission in waiting rooms, individuals with disabilities felt it was hard to convey their concerns and symptoms over the phone. Sometimes they would leave out information or felt that the physicians were rushing the appointment. Potential solutions to continue in-person medical appointments while maintaining safe practices (e.g., hand hygiene) include allowing patients to wait in their cars at the clinic, spreading patient appointments further apart, or reviving a house-call system where doctors make house calls for minor concerns or regular check-ups.

**Solution 3: Revising current financial assistance programs for individuals with disabilities and unpaid caregivers.** Individuals with disabilities and unpaid caregivers faced financial concerns with regards to the rise in expenses (e.g., higher cost of food and personal protective equipment) and a reduction in income. To mitigate these effects, government assistance for these population groups is warranted. To our knowledge, the Canadian government provided a one-time payment of CAD 600 to account for added expenses faced by individuals with disabilities [36]. Considering the effects of the COVID-19 pandemic have lasted nearly two years, we question whether this payment adequately captures the needs of this population. Additionally, although the Canada Emergency Response Benefit (CERB) was in effect, its support was limited to “employed and self-employed Canadians who were directly affected by COVID-19” [37]. Given our knowledge on the challenges individuals with disabilities faced in terms of employment prior to the pandemic, it is likely some individuals with disabilities were ineligible to apply.

Next, unpaid caregivers may have been eligible for the Canadian Recovery Caregiving Benefit (CRCB). Some of the eligibility criteria include individuals who (1) “are caring for their child under 12 years old or a family member who needs supervised care because of their school or care facility being closed or unavailable to them due to COVID-19” and (2) “earned at least $5000 in 2019, 2020 or within the 12 months prior to applying”. However, caregivers who wished to keep their child home from school because of not feeling safe (e.g., fear of contracting COVID-19) would not have been eligible for this benefit [38]. Thus, we question whether this program is comprehensive and inclusive enough given the complex decisions families with disabilities face.

A more comprehensive financial support mechanism is needed to ensure ongoing support for individuals with disabilities for the duration of the pandemic, and consideration should be given to widen the scope of those eligible for the CRCB.

**Solution 4: Improving communications of available support programs.** Many organizations and services, both official and unofficial, cropped up during the pandemic to support individuals with disabilities and other people isolating at home. For example, supports for older adults and individuals with disabilities in Ontario include “Accessible Drive to Vaccines” and the “Ontario community support program” [39]. Similarly, opportunities to learn more about caregiving organizations, or tips on how to support people with dementia, are provided on the Canadian government webpage [40]. However, based on our survey results, it appeared participants were not aware of services in their communities. There is a need to improve the outreach and communications of these groups, to ensure that the people most in need are aware of the services that exist. This includes making a concerted effort to share this information through the mail, community newsletters, local malls, television advertisements, social media, and more.

### 4.4. Strengths and Limitations

This study attempted to take a comprehensive look at the impact of the COVID-19 pandemic and related government precautionary measures on the lives of Canadians living with disability and unpaid caregivers. In doing so, this study asked participants about all facets of daily living, including health, finances, personal relationships, and recreational activities. In maintaining a broad scope, this study helped identify many areas of daily life that have been impacted, and some of the relationships that exist between these areas. Because of online recruitment activities, we were able to recruit participants from across Canada, further contributing to the broad scope of this study. Finally, this study surveyed both individuals with disabilities and unpaid caregivers, allowing for a unique perspective when cross-examining both data sets. As a result, we were able to identify challenges that were similar in both population groups, highlighting areas where potential solutions will be most impactful and beneficial.

This study had several limitations. No personal identifying information was collected as part of the survey to maintain participant anonymity, which could have led to participants completing the survey numerous times, thus biasing the results. Next, this was a web-based survey with advertisements occurring primarily through paid Facebook advertisements. This meant that only eligible individuals with access to the Internet and Facebook would have been able to participate. Other organizations representing individuals with disabilities and unpaid caregivers were contacted about the study; however, we were unsuccessful in creating relationships for recruitment. Because the data of this survey were also collected during lockdown in Ontario, we were limited in our ability to recruit in person. Additionally, because this study was a survey design, we were unable to delve deeper into participant stories to better understand *why* they had certain feelings. This meant that, in instances where participants were vague in their responses, we were unable to probe them further for elaboration of their story. Furthermore, a total of 111 participants were included in this study (43 unpaid caregivers and 68 individuals with disabilities). This prohibited us from completing statistical analyses and could impact the generalizability of these results, as some knowledge saturation was likely not achieved. Lastly, many respondents self-reported as Caucasian, resulting in a lack of diversity in the participant group.

## 5. Conclusions

The results of this study highlight the challenges faced by individuals with disabilities and unpaid caregivers during the COVID-19 pandemic in Canada. Based on these results, we then identified potential solutions that future research should evaluate to mitigate or reduce the negative effects of a pandemic or future emergencies for both population groups.

Specifically, future research should consider: (1) the impact of at-home hand hygiene systems on the transmission of COVID-19 and other viruses during formal and informal caregiving services, (2) the impact of in-person medical appointments on virus transmission, (3) whether increased advertising of support services alleviate stresses experienced by individuals with disabilities and unpaid caregivers, and (4) whether increased government financial supports can reduce the financial stresses experienced by individuals with disabilities and unpaid caregivers during pandemics. In addition to these identified solutions, it is imperative for researchers to work alongside individuals with disabilities and unpaid caregivers and other stakeholders to develop and evaluate practical solutions to address the challenges identified in this work.

## Figures and Tables

**Table 1:** Descriptive statistics for our two groups of participants.

Description	Individuals with Disabilities n (%)	Unpaid Caregivers n (%)
*Sex*	n = 66	n = 42
Male	10 (15.15%)	5 (11.90%)
Female	51 (77.23%)	36 (85.7%)
Other	4 (6.06%)	1 (2.38%)
I prefer not to answer	1 (1.515%)	0 (0.00%)
*Age*	n = 67	n = 42
18–30 years	13 (19.40%)	3 (7.14%)
31–50 years	28 (41.79%)	24 (57.1%)
51–70 years	23 (34.33%)	12 (28.6%)
71–90 years	3 (4.48%)	3 (7.14%)
90+ years	0 (0.00%)	0 (0.00%)
*Household Income*	n = 67	n = 42
<$30,000	40 (59.70%)	12 (28.6%)
$30,000–59,999	14 (20.90%)	10 (23.8%)
600,000–89,999	4 (5.97%)	7 (16.67%)
90,000–119,000	5 (7.46%)	4 (9.52%)
>120,000	0 (0.00%)	4 (9.52%)
I prefer not to answer	4 (5.97%)	5 (11.90%)
*Government Assistance*	n = 68	n = 43
Yes	42 (61.76%)	14 (32.6%)
No	25 (36.76%)	28 (65.1%)
I prefer not to answer	1 (1.47%)	1 (2.33%)

**Table 2:** Participant level of functioning for individuals with disabilities.

Level of Functioning—Brief Model of Disability	Individuals with Disabilities n (%)
*How much of a problem is walking 1 km for you?*	n = 65
1 (None)	13 (20.0%)
2	4 (6.15%)
3	12 (18.46%)
4	8 (12.31%)
5 (Extreme)	27 (41.5%)
6 (N/A)	1 (1.538%)
*How much of a problem is getting where you want to go for you?*	n = 65
1 (None)	9 (13.85%)
2	10 (15.38%)
3	13 (20.0%)
4	14 (21.5%)
5 (Extreme)	18 (27.7%)
6 (N/A)	1 (1.538%)
*How much of a problem is being clean and dressed?*	n = 65
1 (None)	12 (18.46%)
2	18 (27.7%)
3	20 (30.8%)
4	10 (15.38%)
5 (Extreme)	5 (7.69%)
6 (N/A)	0 (0.00%)
*How much of a problem is toileting?*	n = 65
1 (None)	37 (56.9%)
2	13 (20.0%)
3	7 (10.77%)
4	5 (7.69%)
5 (Extreme)	3 (4.62%)
6 (N/A)	0 (0.00%)
*How much of a problem is looking after your health, eating well, exercising or taking your medicines?*	n = 65
1 (None)	
2	5 (7.69%)
3	14 (21.5%)
4	16 (24.6%)
5 (Extreme)	18 (27.70%)
6 (N/A)	12 (18.46%)
	0 (0.00%)
*How much of a problem is feeling tired and not having enough energy?*	n = 65
1 (None)	
2	1 (1.538%)
3	5 (7.81%)
4	9 (14.06%)
5 (Extreme)	16 (25.0%)
6 (N/A)	31 (48.4%)
	3 (4.69%)
*How much of a problem is coping with all the things you have to do?*	n = 65
1 (None)	1 (1.538%)
2	6 (9.23%)
3	11 (16.92%)
4	23 (35.4%)
5 (Extreme)	23 (35.4%)
6 (N/A)	1 (1.538%)
*How much of a problem is remembering to do the important things in your day-to-day life?*	n = 65
1 (None)	
2	4 (6.15%)
3	12 (18.46%)
4	17 (26.2%)
5 (Extreme)	15 (23.1%)
6 (N/A)	16 (24.6%)
	1 (1.538%)
*How much of a problem do you have with getting your household tasks done?*	n = 65
1 (None)	
2	1 (1.54%)
3	5 (7.69%)
4	17 (26.2%)
5 (Extreme)	19 (29.2%)
6 (N/A)	23 (35.4%)
	0 (0.00%)
*How much of a problem do you have with joining community activities (i.e., festivities, religious or other activities)?*	n = 65
1 (None)	
2	1 (1.538%)
3	7 (10.77%)
4	8 (12.31%)
5 (Extreme)	10 (15.38%)
6 (N/A)	34 (52.3%)
	5 (7.69%)
*How much of a problem is using public or private transportation?*	n = 65
1 (None)	9 (13.85%)
2	7 (10.77%)
3	10 (15.38%)
4	16 (24.6%)
5 (Extreme)	16 (24.6%)
6 (N/A)	7 (10.77%)

**Table 3:** Quotes from individuals with disabilities as they relate to each theme.

Theme	Quotes
Individual well-being	
Decline in physical health	“I have had trouble keeping up with oral health, I go week’s [sic] without brushing my teeth. I don’t shower as often and I’m far more sedentary”. “Without physio I’ve backslid a lot and am much weaker and in a lot more pain than before” “I have more pain because I am less active so again don’t get dressed. Hard to stand long enuf to make a meal”
Negative impact to mental health	“I’ve been suicidal again after a long time of doing better and I would attribute that mainly to the restrictions and stress that COVID has brought to my life”. “I have started to use recreational drugs to manage loneliness”
Revising budget	“Every thing is costing so much more on a fix income it is very hard to get food and pay bills and rent”. “Cannot afford take out so sometimes don’t eat. Sometimes not [enough] money for grocery [ies] so don’t eat”. “Greatky [sic] reduced wages and hours to a point the position no longer available was laid off”. “Financially it is hard. Frustrating to see how much CERB was and then how much government expects seniors to live on”. “Husband had part time employment stopped because of COVID. Our income from govt pension does not cover our expenses. We have had to use the Food bank 7 times this year. First time in our lives”.
Changes in accessibility	
Essential needs	“Doctors have only done phone consults. I would forget to mention things on the phone. My pain has increased a lot and I haven’t had the same support before surgery. My health has definitely been impacted as I should have had surgery months ago” “Cannot just walk in a see my doctor anymore have to do facetime which is ridiculous especially if you have lumps how can they see and feel over facetime?” “Yes, I’ve been unable to access care as doctors assume I have access to high speed internet to do video appointments when I don’t have access to high speed internet”. “Impacted grocery shopping (I rely on caregiver sending me pictures and communicating with me through shopping, I now have to order online, lots of changes and missing products.
Leisure activities	“I used to be able to go swimming every Saturday, but COVID restrictions shut down the only wheelchair accessible pool I know of in my ci [sic]” “Cannot go out anywhere. Walk from bed to chair and just sit all day. Fall asleep in chair out of boredom. “I used to go to classes and also was in craft groups which are no longer happening so what I find enjoyable has been taken away from me”. “can’t join any groups that would be fun because everything is closed down and even if it was open I am too scared because everyone else doesn’t care about COVID safety precautions nearly as much as I do”. “Only one good thing came from COVID and that was trying to learn new things to keep busy. Having memory issues learning new things is a challenge but I’ve learned a couple things that I couldn’t do before”.

**Table 4:** Quotes from unpaid caregivers as they relate to each theme.

Theme	Quotes
A loss of income	“The restaurant I worked at before has shut its doors”. “Less pay due to some part time work at a studio that no longer offers the classes I teach”. “Now I’m no longer paid because my caregiving is now full time” “He’s home schooling this year due to autism and he can’t follow health guidelines at all at school ie: mask wearing and staying 2 feet from others and wash his hands etc. So increased caregiving equals decreased hours for work“.
Dual roles: Income earner and caregiver	“I can only work around his day program hours as there’s no other alternative care for him” “I have commited [sic] 80% of my down time to the person I care for so I dont have time to do even a part time job”.
Inability to rest and recharge	“No self care....only priority is husbands care”. “Lack of respite care available which means less time for chores, cleaning and leisure activities”
Mental burden	
Impact to my relationships	“I have only seen 7 family members and no friends since March. I don’t see people because I cannot afford to take COVID to my loved one”. “Relationship with my husband. It’s been very strained and draining” and another participant noted “My children are suffering socially and take out their frustration on me”.
Personal stresses and anxieties	“I get stressed trying to get her help because I am not allowed to go into the ER or to appointments with her and doctors are not getting full answers because she cannot give them. Some nurses have been great while others have been down right rude and completely uncaring”. “Increased stress and anxiety and sucial [sic] thoughts due to financial hardship due to lack of financial support for the child with disabilities”

## Data Availability

Not applicable.
